# Comparative analysis of cattle (*Bos taurus*, 2*n* = 60) and river buffalo (*Bubalus bubalis*, 2*n* = 50) genome assemblies reveals two evolutionary conserved inversions and invalid centromere–telomere orientation of some autosomes

**DOI:** 10.1111/age.70031

**Published:** 2025-07-28

**Authors:** Ramona Pistucci, Ilaria Cascone, Alessandra Iannuzzi, Sara Albarella, Wiktoria Kowal‐Mierzwa, Michele Zannotti, Leopoldo Iannuzzi, Pietro Parma

**Affiliations:** ^1^ Institute for Animal Production System in Mediterranean Environment National Research Council Portici Italy; ^2^ Department of Veterinary Medicine and Animal Production University of Naples Federico II Naples Italy; ^3^ Department of Animal Reproduction, Anatomy and Genomics University of Agriculture in Krakow Krakow Poland; ^4^ Department of Agricultural and Environmental Sciences University of Milan Milan Italy

**Keywords:** cattle, evolution, FISH, karyotype, river buffalo

## Abstract

This study investigates autosome evolution between river buffalo (*Bubablus bubalis*, BBU) and cattle (*Bos taurus*, BTA), two closely related species within the *Bovidae* family. Despite differences in chromosome numbers (2*n* = 60 in cattle and 2*n* = 50 in river buffalo), previous cytogenetic studies have shown high autosome similarity. However, standard banding techniques have limitations in detecting small‐scale genomic rearrangements. Using molecular comparisons, this study identifies two previously undetected chromosomal inversions: a 30‐Mb inversion on BBU7 (compared to BTA6) and a 4‐Mb inversion on BBU14 (compared to BTA13). These findings were validated through bioinformatics analyses (genomic alignments and BLAST searches) and confirmed via fluorescence in situ hybridization technique. In addition, it has been shown that several river buffalo chromosomes are shown inverted in the genome assembly considered in this study (NDDB_SH_1). The study highlights that autosome evolution in *Bovidae* involves not only centric fusions but also cryptic intra‐chromosomal rearrangements. These results contribute to a deeper understanding of genome evolution in closely related species and demonstrate the importance of high‐resolution molecular techniques in uncovering hidden genomic changes.

## INTRODUCTION

The river buffalo (*Bubalus bubalis*) and cattle (*Bos taurus*) both belong to the tribe Bovini, one of the several tribes within the family *Bovidae*. The closest common ancestor of cattle and river buffalo has been traced back to the middle Miocene, approximately 13.2 million years ago (Hassanin et al., 2012, 2013). In terms of genome organization, river buffalo and cattle have different chromosome numbers (2*n* = 50 for *B. bubalis*—river type—and 2*n* = 60 for *B. taurus*), but they share the same autosomal fundamental number (FN = 58). This discrepancy arises from five centric fusions involving 10 acrocentric bovine chromosomes, which resulted in five bi‐armed river buffalo chromosomes (Iannuzzi, 1994; Iannuzzi et al., 2021). In this family, centric fusions have appeared to be the principal mechanism of autosome evolution (Iannuzzi et al., 2009). Except for the five centric fusions, cytogenetic analyses comparing the two karyotypes reveal perfect autosomal similarity between the two species; even in these instances, the homology between river buffalo chromosome arms and cattle chromosome arms is preserved. However, these analyses, conducted using various chromosome banding techniques, have a significant limitation: they cannot detect evolutionary rearrangements below a certain resolution threshold.

A cryptic rearrangement of 7.4 Mb between bovine and goat was identified previously (De Lorenzi et al., 2015), and a 1.5‐Mb rearrangement between cattle and sheep was demonstrated more recently (De Lorenzi et al., 2020). These findings suggest that evolutionary cryptic rearrangements, while present, remain undetectable through standard cytogenetic procedures.

In this study, by comparing the autosomal genomes of river buffalo and cattle at the molecular level, we uncovered two evolutionary rearrangements that had previously gone undetected using standard cytogenetic techniques.

## MATERIALS AND METHODS

### Identification of genome divergences

Genome divergence detection between river buffalo and cattle was conducted following the methodology described by De Lorenzi et al. (2015). The getfasta function in the Bedtools package was used to extract the first 400 bp of each Mb of each river buffalo chromosome (Quinlan & Hall, 2010). The analyses were performed using software available on the Galaxy platform (Afgan et al., 2016). Using the NDDB_SH_1 genome assembly for river buffalo, each 400‐bp sequence was compared with the cow genome (ARS‐UCD1.3 genome assembly) using the NCBI BLAST tool (Altschul et al., 1990; Sayers et al., 2022). The data were then visualized in Excel, with each point representing sequence locations in the two different genomes.

### Cytogenetic analysis

The bacterial artificial chromosomes used to generate the probes (Table 1) are all from the CHORI‐240 library, and the fluorescence in situ hybridization (FISH) studies were performed in accordance with De Lorenzi and Parma (2020).

**TABLE 1:** Characteristics of bacterial artificial chromosomes used for fluorescence in situ hybridization probe production.

Name	BTA	Position^a^ (Mb)	END sequences ^b^
346 J14	BTA6	10.6	CC505807/CC505720
437 J1	BTA6	30.7	CC552074/CC551985
386G19	BTA13	13.2	CC588008/CC587923
421B5	BTA13	16.2	CC540860/CC540765

Abbreviation: BTA, *Bos taurus*.

^a^ Position on ARS_UCD_1.3 genome assembly.

^b^ End sequence of the bacterial artificial chromosome available on GenBank.

### Long read sequence analyses

Data from two whole‐genome sequencing projects were used to confirm differences between the two genomes: (i) genomic sequences obtained using Oxford Nanopore technology (Deamer et al., 2016) for river buffalo; and (ii) PacBio HiFi technology (Travers et al., 2010) for cattle. Sequences were visualized using Integrative Genomics Viewer (Robinson et al., 2011), and BLAST was used to search for implicated sequences. Regions containing proximal and distal potential evolutionary breakpoints (EBPs) were manually analyzed (Altschul et al., 1990).

## RESULTS

**FIGURE 1:**
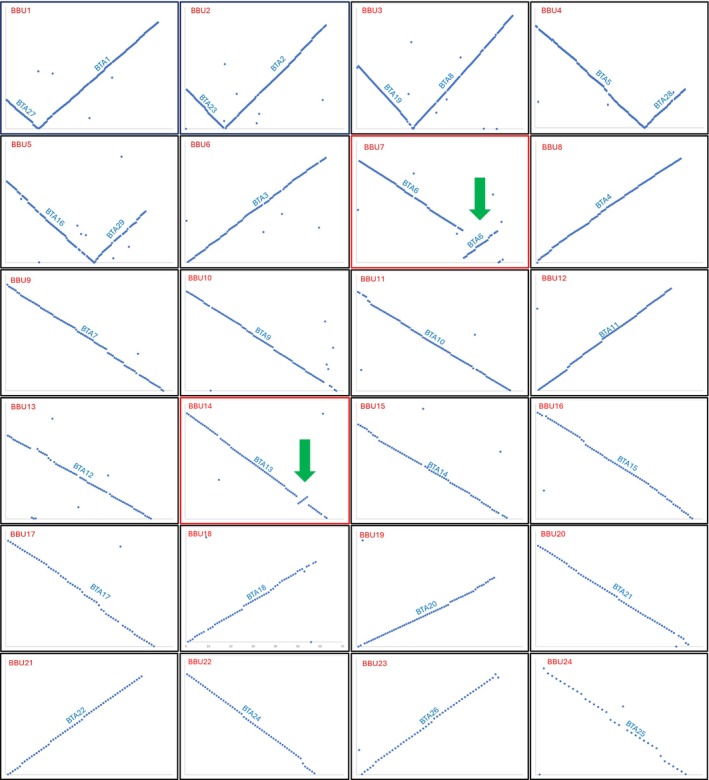
Cattle (BTA) vs. river buffalo chromosome (BBU) genomic alignments. Genomic alignments between cattle and river buffalo chromosomes are shown. Each dot represents a fragment mapped onto the two genomes. Two contiguous points are 1 Mb apart. The river buffalo genome is represented on the *X*‐axis, the cattle genome on the *Y*‐axis. The two potential inversions are highlighted in red. A positive slope of the straight line identified by the dots indicates that the river buffalo chromosomes are correctly reported in the genome assembly; conversely, a negative slope indicates an inverted view. Green arrows indicate the two inversions identified.

The genomic sequence alignments between river buffalo and cattle autosomes are shown in Figure 1. Anomalies were defined as cases where at least three consecutive probe sequences were out of alignment (represented by three dots on the graphs), deviating from the expected perfect chromosomal alignment. Single dots, present in almost all chromosomes, represent river buffalo sequences mapping onto cattle genomic unmapped fragments. One exception is the three BBU13 fragments, all of which map to a large cattle unmapped fragment rather than to BTA12.

One key factor examined was chromosome orientation. Some chromosomes were found to be inverted, meaning that bp position 1 indicated the telomeric region rather than the centromeric region. This finding is crucial, as it can affect the selection of potential centromeric or telomeric genomic probes. The chromosomes involved include: BBU4, BBU5, BBU7, BBU9, BBU10, BBU11, BBU13, BBU14, BBU15, BBU16, BBU17, BBU20, BBU22, and BBU24. Regarding the five river buffalo chromosomes with 2 arms (BBU1, 2, 3, 4, and 5), only the last two are reported in the reverse direction. In this case, base number 1 corresponds to the telomere of the long chromosome of the pair and not to that of the short chromosome as it should be. These inversions were confirmed using previously available physical mapping data (data not shown).

**FIGURE 2:**
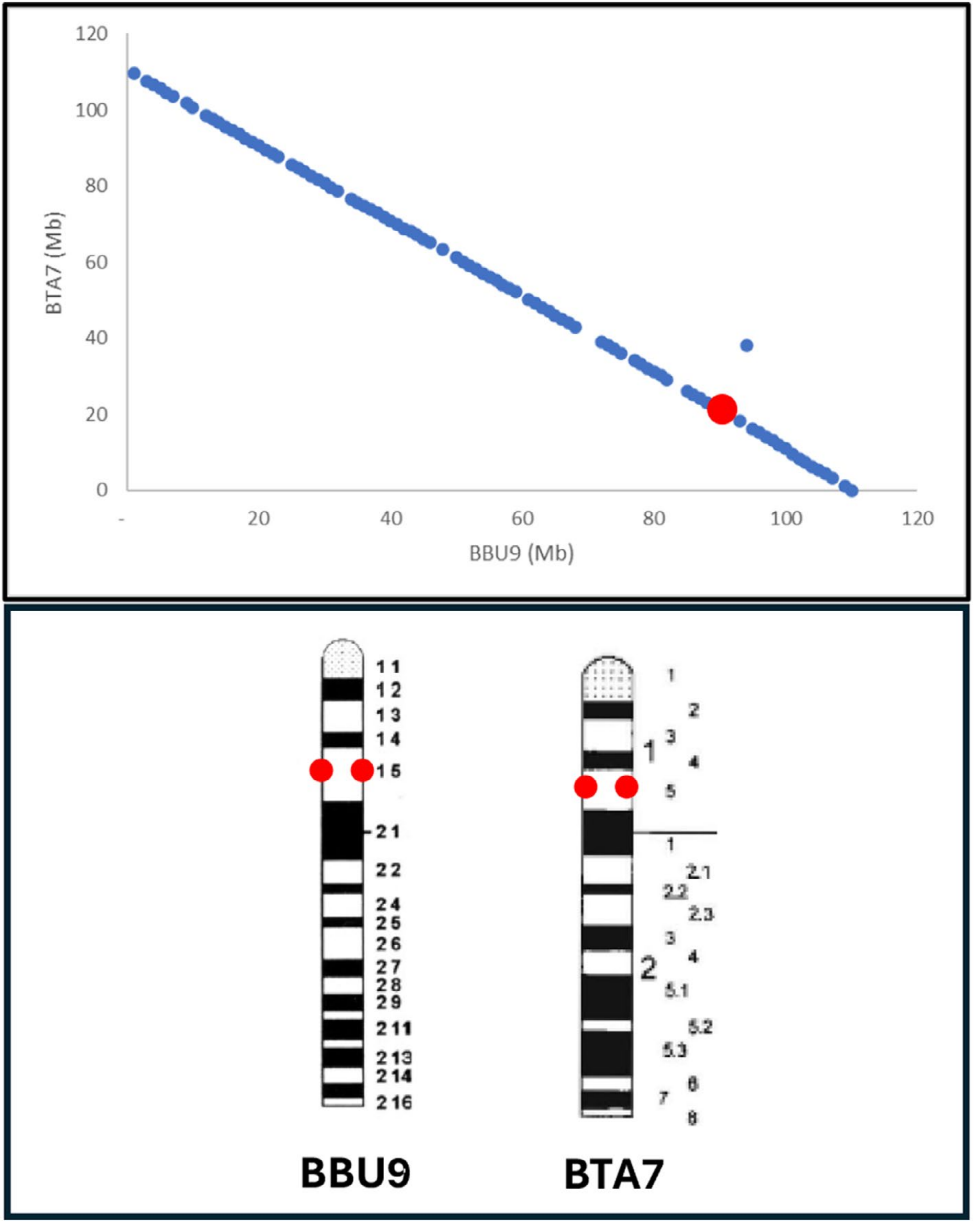
Verification of river buffalo chromosome orientation. The physical localisation of the VAV1 gene in cattle and river buffalo obtained by fluorescence in situ hybridization is reported (Di Meo et al., 2008). Chromosome ideograms are derived from published standard karyotypes: ISCNDB 2000 (2001) (cattle) and Iannuzzi (1994) (river buffalo). BBU9, river buffalo chromosome 9; BTA7, cattle chromosome 7.

An example is reported for BBU9, where the VAV1 gene is located at 93.5 Mb. Given that BBU9 is 110 Mb long, VAV1 should reside in the telomeric region. However, FISH mapping in both species places VAV1 near the centromere (Di Meo et al., 2008), confirming the inversion of BBU9 (Figure 2).

**FIGURE 3:**
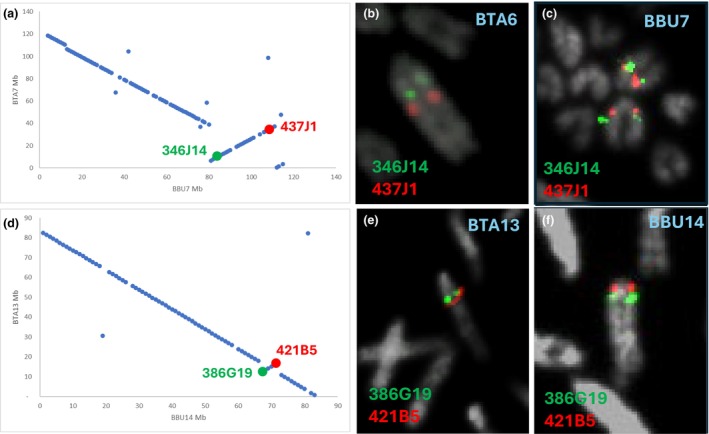
Analysis of inversions by fluorescence in situ hybridization (FISH). The two inversions found by bioinformatics analysis were confirmed by FISH, which are reported. (a, d) Bio‐informatic results. (b, e) FISH results on cattle metaphase. (c, f) FISH results on river buffalo metaphase. The colors used to indicate the bacterial artificial chromosomes used as probes (green and red) are representative of the FISH probes on the metaphases.

Looking at the alignments between river buffalo and cattle chromosomes, two significant anomalies can be seen: an approximately 30‐Mb inversion of BBU7 compared to its bovine counterpart BTA6 (Figure 3a) and a smaller (around 4 Mb) inversion of BBU14 compared to its counterpart BTA13 (Figure 3d). These inversions between river buffalo and cattle were confirmed by FISH (Figure 3b,c,e,f).

**FIGURE 4:**
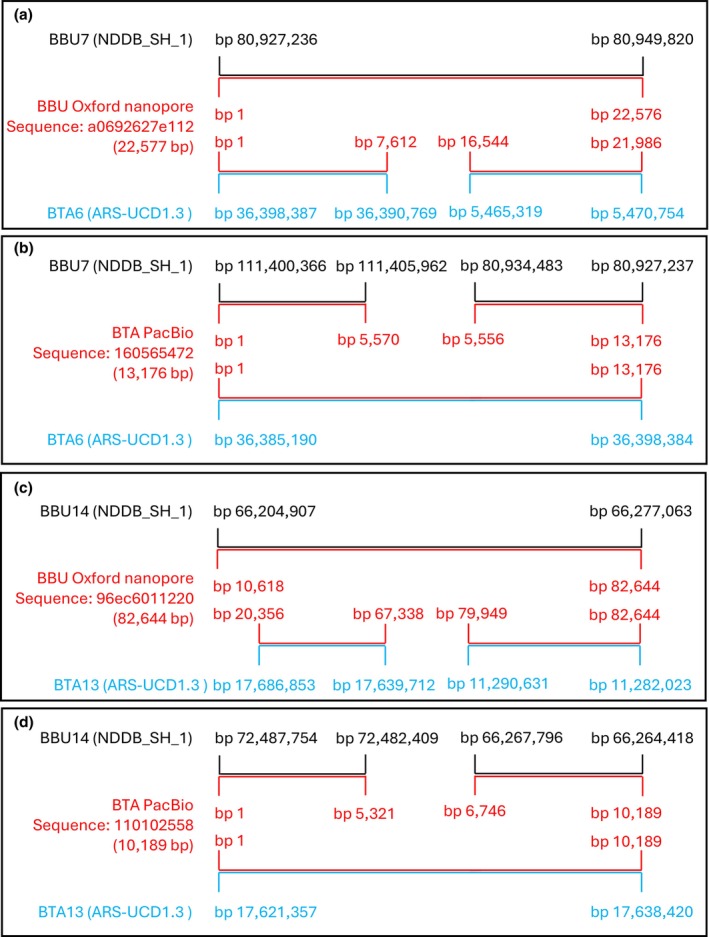
Breakpoint analysis by means of long‐read sequences. The figure shows the alignments of 4 long read sequences with the cattle and river buffalo genomes. (a) The river buffalo genome sequencing produced the sequence a0692627e112, which matches perfectly with BBU7 but breaks when matched with the BTA6. (b) Cattle genome sequencing produced the sequence 160565472, which matches perfectly with BTA6 but breaks when matched with the BBU7. (c) The river buffalo genome sequencing produced the sequence 96ec6011220, which matches perfectly with BBU14 but breaks when matched with the BTA13. (d) Cattle genome sequencing produced the sequence 110102558, which matches perfectly with BTA13 but breaks when matched with the BBU14.

Long‐read sequencing further validated the presence of these inversions. In cases of inversion, a river buffalo sequence located near an EBP should align perfectly with the river buffalo genome but appear “broken” in the bovine genome. This pattern was confirmed using BLAST for both the BBU7/BTA6 (Figure 4a,b) and BBU14/BTA13 (Figure 4c,d) inversions.

**FIGURE 5:**
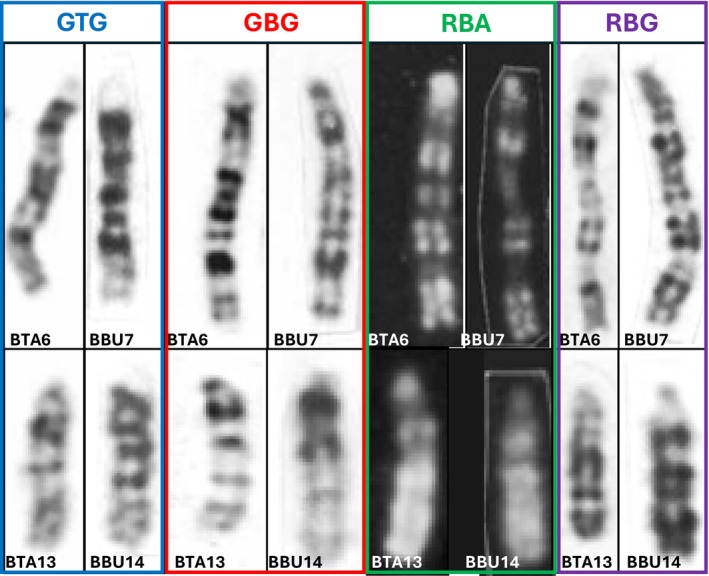
Comparative analysis of chromosomes involved in inversions. The figure shows the comparison between BTA6 and BBU7 and between BTA13 and BBU14 with four different banding techniques. The types of banding and classification of chromosomes are shown directly in the figure. In details they are: GBG, G‐banding by early BrdU‐incorporation and Giemsa staining; GTG, G‐banding by trypsin and Giemsa‐staining; RBA, R‐banding by late BrdU‐incorporation and Acridine orange staining; RBG, R‐banding by late BrdU‐incorporation and Giemsa staining. The chromosomes reported are obtained and elaborated from Iannuzzi (1996) for cattle and Iannuzzi (1994) for river buffalo.

G‐ and R‐banding comparisons between cattle and river buffalo chromosomes involved in the two inversions (BTA6/BBU7 and BTA13/BBU14) revealed striking similarities (Figure 5).

## DISCUSSION

The presence of cryptic chromosome evolution in phylogenetically close species has been previously demonstrated. The findings of this study confirm that river buffalo and cattle also exhibit such rearrangements, reinforcing the notion that chromosome evolution in the *Bovidae* family occurs not only through centric fusions but also through specific intra‐chromosomal rearrangements. Notably, standard banding techniques did not reveal these inversions. In the case of BTA6/BBU7, the inversion involves the first two centromeric bands, preserving the overall banding pattern. The BTA13/BBU14 inversion, involving only 3 Mb, is similarly undetectable via banding.

Chromosomal rearrangements can sometimes lead to significant phenotypic changes by modifying gene expression. For example, in pigs, a genetic component affecting salt perception (SCNN1B) was found in a species‐specific EBP, impacting salt taste sensitivity (Groenen et al., 2012).

The EBPs identified in this study are approximately located at 36 390 769 and 5 465 319 bp for BTA6, and 17 639 712 and 11 290 631 bp for BTA13. The interference of an EBP with the function of a genetic factor can occur not only through disruption of the factor itself but also through interference with its regulatory region. This aspect complicates the ability to predict the effect of an EBP, as precisely defining the size of a regulatory region is challenging. There are several examples showing that regulatory elements can be located several kilobases away from the transcription start site. One such example is the SOX9 gene: breakpoints located more than 130 kb from the gene cause campomelic dysplasia in human patients (Wirth et al., 1996). More recently, a regulatory element for the Sry gene has been hypothesized to lie approximately 84 kb upstream of the transcription start site (Dupont et al., 2025).

As for the four EPBs identified in this study, their position relative to nearby genetic factors varies from case to case. The two EBPs on BTA6 do not directly involve any genetic factor; among these, only the one at position 36 390 769 is located upstream at a reasonable distance from two genes: HERC6 (−25 kb) and PPM1K (−60 kb).

Regarding the EBP located on BTA13, the one at position 11 290 631 appears to lie within a poorly characterized genetic factor: LOC112449367, ankyrin repeat domain‐containing protein 62‐like. However, identifying this genetic factor in the river buffalo assembly is complex, as there are multiple locations on BBU14 with varying orientations.

Finally, the EBP on BTA13 at position 17 639 712 involves the 5′ untranslated region of another poorly characterized genetic factor (LOC112449258), which is also an ankyrin repeat domain‐containing protein like.

In conclusion, this study identifies two chromosome inversions between river buffalo and cattle. Both bioinformatics and FISH analyses confirm these rearrangements, which appear to have no direct association with known genetic factors. It was also shown that 14 river buffalo chromosomes are shown inverted in the genome assembly considered in this study.

## AUTHOR CONTRIBUTIONS

Ramona Pistucci: cytogenetic investigation. Ilaria Cascone: genomic investigation. Alessandra Iannuzzi: cytogenetic investigation, data curation. Sara Albarella: genomic investigation, data curation. Wiktoria Kowal‐Mierzwa: cytogenetic investigation. Michele Zannotti: cytogenetic investigation. Leopoldo Iannuzzi: cytogenetic investigation, review and editing. Pietro Parma: conceptualization, data interpretation, writing and editing.

## FUNDING INFORMATION

This study has been partially supported by the project PON01_486 GENOBU, Ministry of Education, University and Research.

## CONFLICT OF INTEREST STATEMENT

The authors declare no conflicts of interest.
